# Synthesis of Multi-Walled Carbon Nanotubes from Plastic Waste Using a Stainless-Steel CVD Reactor as Catalyst

**DOI:** 10.3390/nano7100284

**Published:** 2017-09-22

**Authors:** Pranav K. Tripathi, Shane Durbach, Neil J. Coville

**Affiliations:** DST-NRF Centre of Excellence in Strong Materials and the Molecular Sciences Institute, School of Chemistry, University of the Witwatersrand, Johannesburg 2050, South Africa; Shane.Durbach@twu.ca

**Keywords:** plastic waste, multi-walled carbon nanotubes, upgrading of plastic waste, two-stage self-catalytic CVD reactor, dusting catalyst

## Abstract

The disposal of non-biodegradable plastic waste without further upgrading/downgrading is not environmentally acceptable and many methods to overcome the problem have been proposed. Herein we indicate a simple method to make high-value nanomaterials from plastic waste as a partial solution to the environmental problem. Laboratory-based waste centrifuge tubes made of polypropylene were chosen as a carbon source to show the process principle. In the process, multi-walled carbon nanotubes (MWCNTs) were synthesized from plastic waste in a two-stage stainless steel 316 (SS 316) metal tube that acted as both reactor vessel and catalyst. The steel reactor contains Fe (and Ni, and various alloys), which act as the catalyst for the carbon conversion process. The reaction and products were studied using electron probe microanalysis, thermogravimetric analysis, Raman spectroscopy and transmission electron microscopy and scanning electron microscopy. Optimization studies to determine the effect of different parameters on the process showed that the highest yield and most graphitized MWCNTs were formed at 900 °C under the reaction conditions used (yield 42%; Raman *I*_D_/*I*_G_ ratio = 0.48). The high quality and high yield of the MWCNTs that were produced in a flow reactor from plastic waste using a two stage SS 316 chemical vapor deposition (CVD) furnace did not require the use of an added catalyst.

## 1. Introduction

Plastic waste (mainly made of polymeric carbon-containing materials) is widely considered as a source of air, soil, water and marine pollution [1,2,3,4]. Indeed most industrial and household plastic commodities made using virgin plastic or recycled plastic waste are non-biodegradable and their disposal thus has an irreversible environmental impact on land, air and water [5]. In the extreme, Rochman et al. have advocated classifying plastic waste as a hazardous material so that its disposal and reusability can be ensured [6]. The lifespan of plastic waste, (e.g., polypropylene) on earth can be thousands of years before biodegradation or oxidation is complete [7,8]. Thus, it is necessary to either economically recycle plastic waste or to find alternative solutions to remove it from the environment. Most importantly, the huge amounts of plastic waste generated can also be viewed as a potential hydrocarbon resource.

Carbon nanomaterials (CNMs) such as carbon nanotubes (CNTs), carbon nanofibers (CNFs) and carbon spheres (CSs) are materials which have remarkable physical and chemical properties, that include their strength and electrical conductivity [9], but their production processes are energy and resource intensive [10,11,12,13,14,15,16,17]. In recent years, researchers have suggested using plastic waste as a carbonaceous feedstock to make CNTs, CNFs and CSs [18,19,20,21,22,23,24]. The production of multi-walled carbon nanotubes (MWCNTs) from polymers has been found to be achievable through a variety of different catalytic and thermal methods in autoclaves, by chemical vapor deposition (CVD), in quartz tube reactors, muffle furnaces, and fluidized beds, etc. [18,20,25]. Indeed, the CVD method is one of the most successful methods that has been used to make MWCNTs on an industrial scale [20,26,27].

In recent years a number of reports on the direct growth of MWCNTs using in-situ methods of synthesis have been published, in which carbon containing species are passed over a transition metal alloy that acts as a catalyst. The synthesis of the catalyst can, however, be very tedious, lengthy and un-economical and can increase the cost of the MWCNTs. Many studies have also shown that stainless steel (SS) can act as a catalyst to synthesize MWCNTs and CNFs [28,29,30,31,32,33]. 

The unintentional reaction of a steel reactor with carbon containing materials (e.g., hydrocarbons) leads to the corrosion of the steel reactor surface and eventually to the rupture of the reactor with severe safety consequences. Many studies have thus been performed to avoid this corrosion process. The corrosion process entails a steel-surface-carbon interaction in which the metal surface atoms are removed to yield a pitted steel surface. This process is known as dusting [34]. In the dusting process both CNTs and CNFs are formed. It is a process in which the steel chromium over-layer is removed to expose the underlying metals (e.g., Fe, Ni) which act as catalysts for carbon growth [29,30]; in industry, much research has been expended in understanding and preventing dusting. 

In this study, we have exploited and made positive use of the concept of dusting and combined it with the problem of plastic waste removal to provide a method for making CNTs cheaply from a model polymer, polypropylene (PP). More specifically we have made MWCNTs from used PP centrifuge tubes as a carbon source in a two-stage CVD furnace made of a SS 316 metal tube. The SS 316 tube was used as both the reactor and the catalyst. Thus, no added catalyst was used in the process. The SS 316 tube was chosen because it has a high content of iron and nickel, which are well known catalysts for the synthesis of MWCNTs. Since oxidation or oxidation-reduction processes can modify a steel surface, we have used these reactions to enhance the reaction of the PP with the SS reactor surface [35]. Herein we report on the synthesis, optimization, and characterization of CNTs (and other carbons) made from PP waste in a stainless-steel reactor without an added catalyst.

## 2. Results and Discussion

### 2.1. Synthesis of MWCNTs Using Plastic Waste as a Carbon Feed and an SS 316 Steel Reactor as Catalyst

In a typical CVD synthesis procedure the carbon feed is usually in the form of a gas or a liquid vapour [36]. However, examples are known in which a solid molecular material (e.g., camphor) was used for the synthesis of MWCNTs in the CVD process [37]. In this CVD synthesis we used centrifuge tube plastic waste as an example of a solid plastic waste material, which can be utilized as a carbon feed stock for the synthesis of MWCNTs in a novel process. In a generic CVD process to make CNTs, the catalyst (typically a transition metal) is either placed in the reactor, [38] or the catalyst is added to the reactor in the gas phase (e.g., ferrocene (C_10_H_10_Fe) or Fe(CO)_5_) [39]. In this study we have used a reactor made of steel as the catalyst, i.e., without the use of an additional catalyst. 

**Figure 1:**
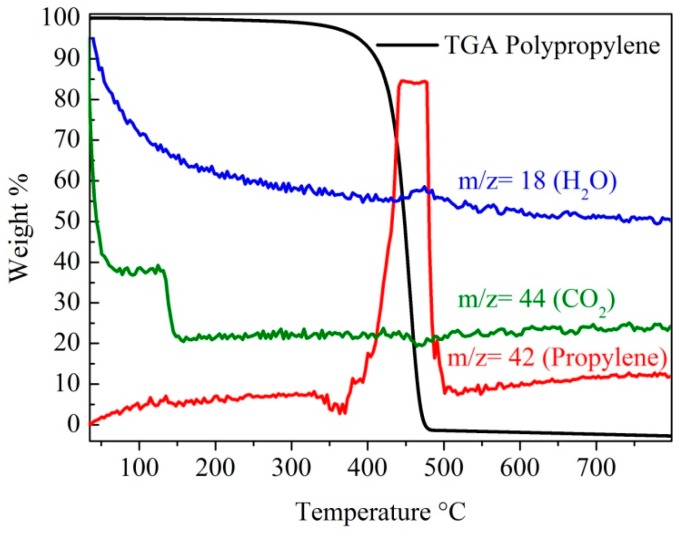
Hyphenated TGA-GC-MS data obtained from the plastic waste centrifuge tubes under a nitrogen gas flow.

In the initial experiments, the reactions to determine the appropriate conditions for the conversion of plastic waste back to propylene were established. The experiments were performed by heating pieces of plastic waste centrifuge tubes at different temperatures under a nitrogen flow and evolved gases were monitored by a hyphenated gas chromatography-mass spectrometry attached to a Thermo-gravimetric analysis (TGA-GC-MS). The weight loss of the plastic waste against temperature showed that the plastic decomposed between 400–500 °C under an inert atmosphere. This temperature range for the decomposition of the PP was thus chosen for the CVD experiments. Further, the mass spectrometry data (Figure 1) confirmed that the waste decomposed almost completely to propylene. During the decomposition of the plastic waste a very small amount of water vapor was also released. No carbon monoxide or any other mass peaks associated with propylene fragments were observed.

The two stage CVD furnace was made of SS 316 metal, which consisted of 45–60% iron, 10–14% nickel, 16–18% chromium and some other transition metals found in very low amounts (Table 1) [40]. Thus, the reactor was rich in both iron and nickel, which are both known to be good catalysts for making MWCNTs [41].

**Figure 2:**
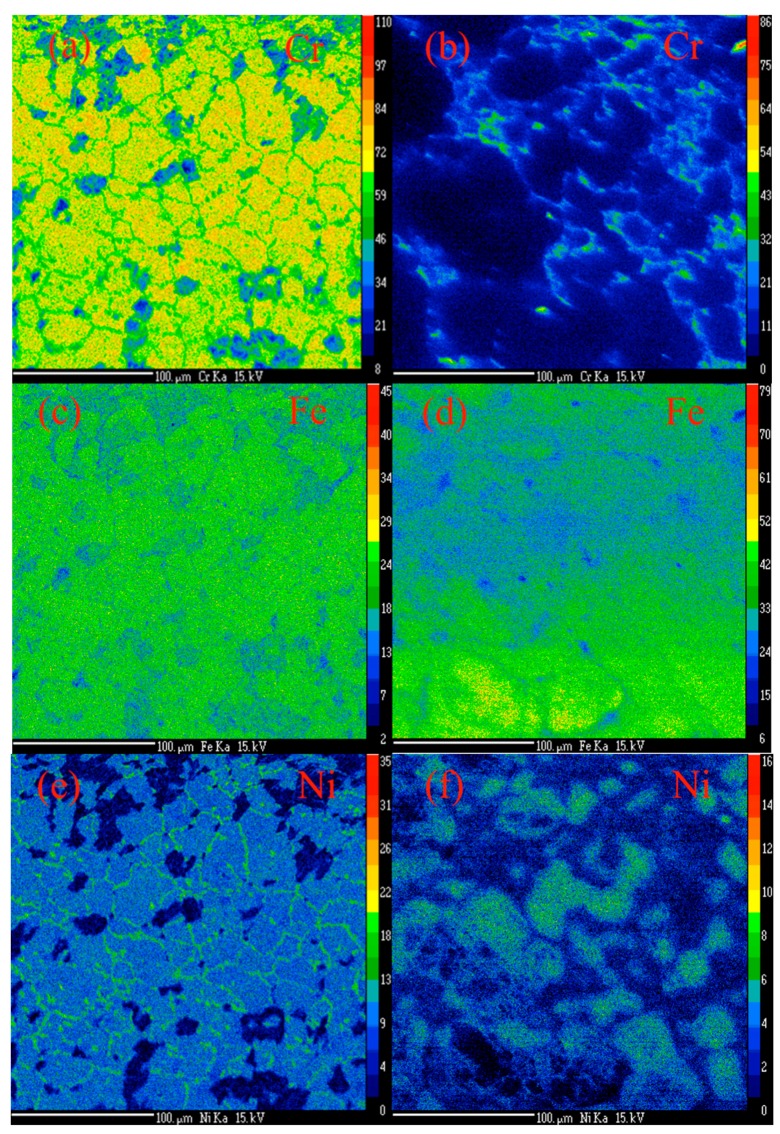

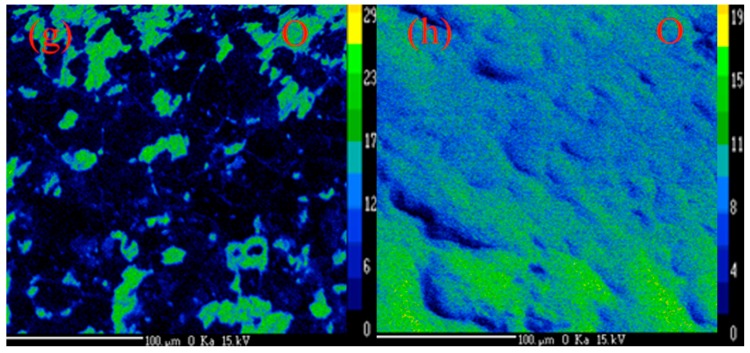
EMPA-WDS element distribution on the steel tube reactor before [(**a**) chromium (Cr-Kα), (**c**) iron (Fe-Kα), (**e**) nickel (Ni-Kα) and (**g**) oxygen (O-Kα)] and after oxidation [(**b**) chromium (Cr-Kα), (**d**) iron (Fe-Kα), (**f**) nickel (Ni-Kα), and (**h**) oxygen (O-Kα)]. The element intensity scale bar is shown on the right-hand side of each element images.

Before a CVD experiment, the steel tube reactor was oxidized in a static air atmosphere at 900 °C for one hour [35]. Thereafter, the reactor was cooled to room temperature and the surface of the tube was removed with a nylon brush. The steel tube both before and after oxidation was characterized by electron probe microanalysis–wavelength dispersive X-ray spectroscopy (EPMA-WDS) (Figure 2). Chromium mapping on the pristine SS 316 metal (Figure 2a) shows that the surface of the tube is, as expected, extensively covered with chromium. It is well know that the chromium is used to protect the steel from corrosion or dusting processes. After oxidation, the chromium has largely been removed/oxidized (Figure 2b) and what was detected was observed to be homogeneously distributed at the grain boundaries. Figure 2c,d shows the iron distribution in the metal tube before and after oxidation respectively. The images reveal substantial iron content in both images, but with the Fe more homogeneously distributed before oxidation. Figure 2e,f shows the mapped nickel particle concentrations on the metal tube before oxidation and after oxidation, respectively. Prior to oxidation the nickel can be seen along grain boundaries while after oxidation the Ni at the grain boundary has disappeared (Figure 2f). As expected, the oxygen on the surface of the steel has increased after oxidation; the images suggest a more pitted structure after the reaction with air (Figure 2g,h).

**Figure 3:**
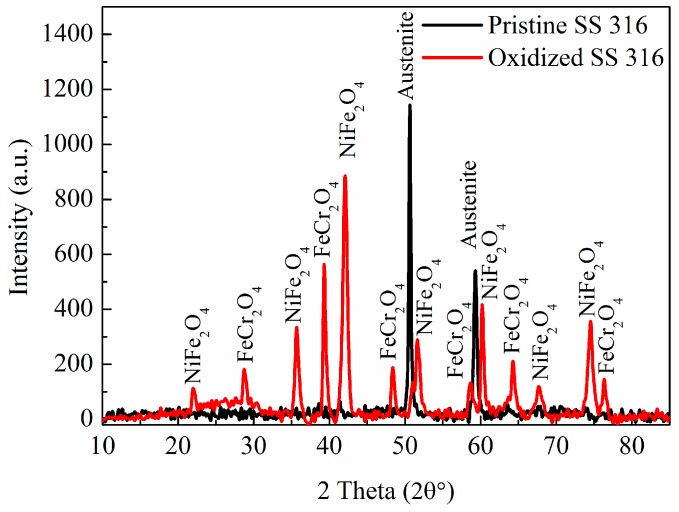
XRD patterns of pristine and oxidized SS 316. The data indicate the transformation of the steel reactor (austenite) structure to nickel iron oxide and iron chromium oxide after reactor oxidation in static air at 900 °C for 1 h.

X-ray powder diffraction (XRD) analysis was also performed on the pristine and oxidized surface of a cut section of the reactor tube (Figure 3). The pristine tube showed the presence of fcc austenite at 2*θ* values of 50.7° (111) and 59.2° (200) with their respective d values of 0.20 and 0.18 nm [42]. After oxidation new peaks could be indexed to iron chromium oxide and nickel iron oxide (2*θ* values and their respective crystal planes for fcc nickel iron oxide occurred at 21.3° (111), 35.5° (220), 41.9° (311), 50.9° (400), 51.7° (111), 67.7° (511) and 74.3° (440) (JCPDS: 00-003-0875) [43] and for rhombohedral iron chromium oxide at 28.3° (012), 39.1° (104), 48.2° (113), 48.4° (024), 58.4° (024), 64.1° (116) and 76.2° (300) (JCPDS: 00-034-0140)) [44].

### 2.2. Morphology, Yield and Crystallinity of the Synthesized MWCNTs

**Figure 4:**
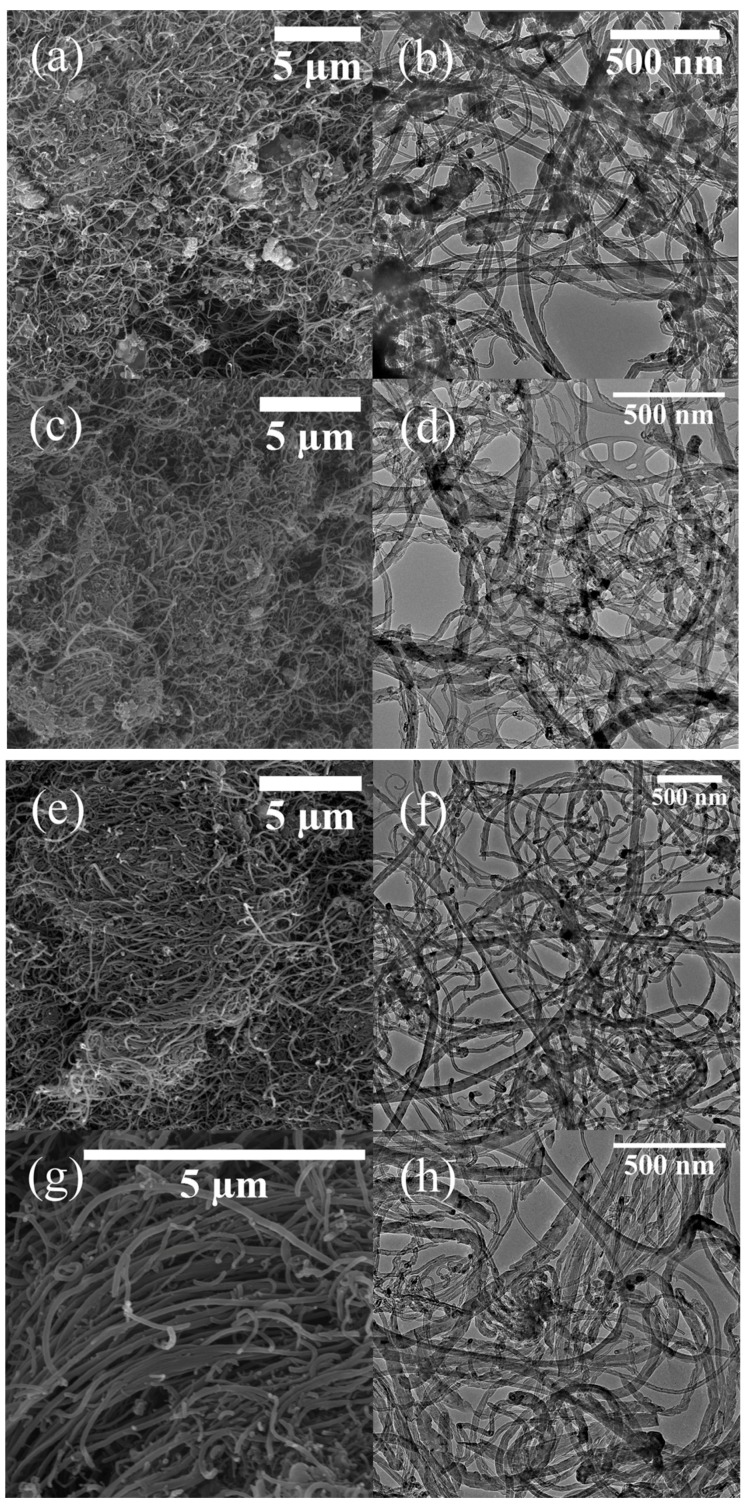

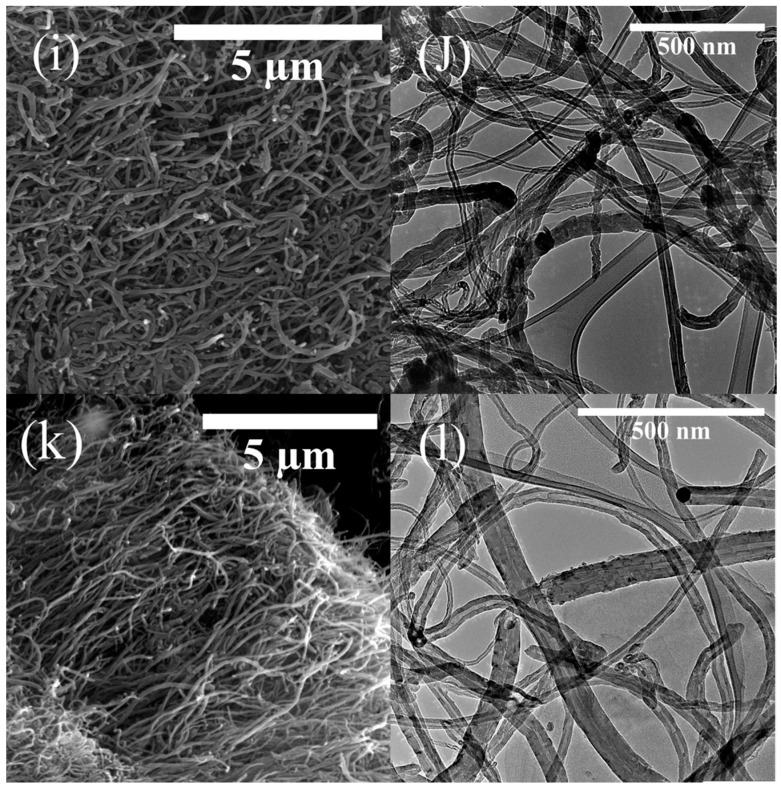
SEM and TEM micrographs recorded on the reaction products. (**a**,**b**) W-MWCNT-600; (**c**,**d**) W-MWCNT-700; (**e**,**f**) W-MWCNT-800; (**g**,**h**) W-MWCNT-900; (**i**,**j**) W-MWCNT-1000 and (**k**,**l**) W-MWCNT-1100.

Scanning electron microscopy (SEM) and transmission electron microscopy (TEM) micrographs of all the carbons grown on the oxidized SS 316 are shown in Figure 4. The SEM images (Figure 4a) show that the synthesized material at 600 °C contained a mixture of impurities, most likely amorphous carbon, CNFs and MWCNTs (no carbon was recovered at temperatures less than 600 °C). As the synthesis temperature was increased to 700 °C the amount of amorphous carbon was reduced (Figure 4c–d) and at 800 °C amorphous carbon formations were not observed (Figure 4e–f).

**Figure 5:**
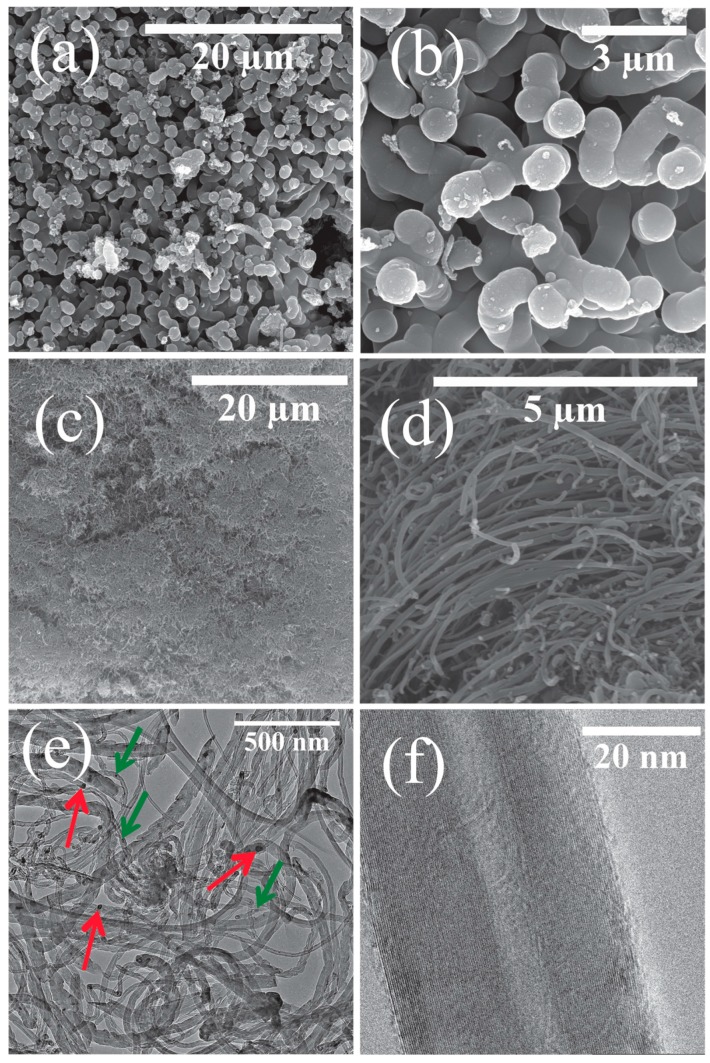
SEM micrographs of CNFs-900 (**a**,**b**) and W-MWCNT-900 (**c**,**d**), TEM micrographs of W-MWCNT-900 at low and higher resolutions are shown in (**e**,**f**). In image (**e**) red arrows show metal in the tip of the MWCNTs and the green arrows shows metal inside the MWCNTs.

A more in-depth analysis of the carbon produced at 900 °C (Figure 5) was undertaken and is discussed below (Figure 5). Carbon nanofibers (CNFs) grown at 900 °C on the non-oxidized SS 316 tube are presented in Figure 5a,b. Under these conditions large CNFs (Figure 5a) and some amorphous carbons were formed. The diameters of the CNFs are ca. 50 nm with lengths of 5–8 µm. These fibers are filled and there was no hollow structure observed from the TEM images (data not presented in this report). The yield of CNFs grown on the non-oxidized steel tube was very poor (ca. 2.3 wt %). EPMA (Figure 2) and XRD (Figure 3) data of the non-oxidized steel reactor showed that chromium dominated the surface. It is known from the literature data that chromium is not a good catalyst for MWCNTs growth [45]. Thus, on the non-oxidized tube, thermal cracking of PP will occur to form only soot, spheres, and amorphous carbon with the morphology and yield determined by the reaction temperature. 

Figure 5c indicates that on the oxidized tube the carbon grown forms a thick mat and the higher resolution (Figure 5d) shows that the mat is made of fibrous carbon materials. The low resolution TEM image of W-MWCNT-900 (Figure 5e) reveals that the carbons are indeed MWCNTs with variable diameters (ca. 20–35 nm). These kinds of MWCNTs were also grown at higher temperature (1000 and 1100 °C) (see Figure 4i–l). In Figure 5e metal can be seen in the tip of the MWCNTs as well as inside the tube. This suggests that the CNT growth process on the metal reactor surface follows a tip growth process. Figure 5f shows a high resolution TEM micrograph of W-MWCNT-900 with well-defined multiwall layers (inner diameter of 10 nm and a tube diameter of 35 nm with a distance between the two tube walls of 0.35 nm) which is typical of MWCNTs [46]. The inner diameters and outer diameters of the MWCNTs are presented in Table 2. The TEM image reveals that the MWCNTs obtained at 900 °C from plastic waste are highly graphitic. Similar morphologies and structures were noted for MWCNTs grown at 1000 and 1100 °C (Figure 4i–l).

**Figure 6:**
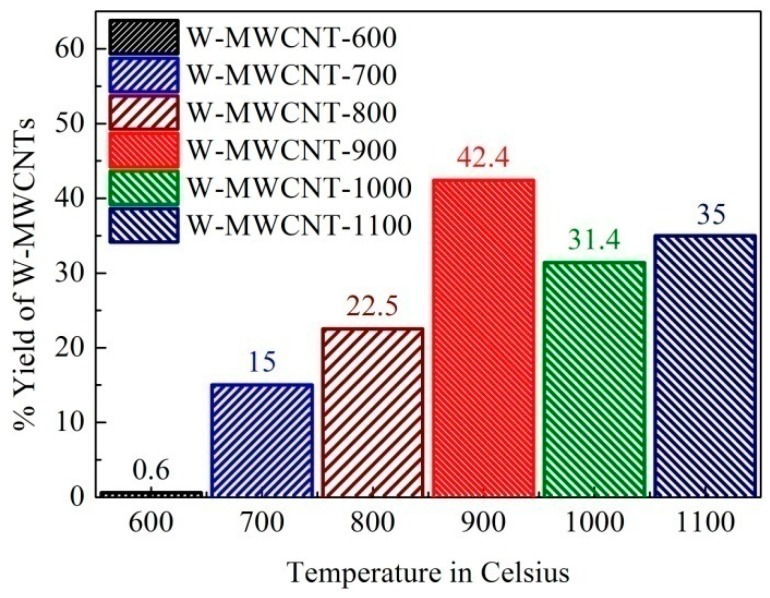
Yield of MWCNTs synthesized from plastic waste without the use of an external catalyst.

The yield of MWCNTs synthesized at the different reactions temperatures was calculated by using Equations (1) and (2) and the data are presented in Figure 6. As the synthesis temperature increased, the yield increased. At 800 °C a good yield of MWCNTs (22.5%) was recovered while at 900 °C the highest yield (42.4%) was obtained. A further increase in temperature (1000 and 1100 °C) led to reduced yields (31.3% and 35% respectively). 

**Figure 7:**
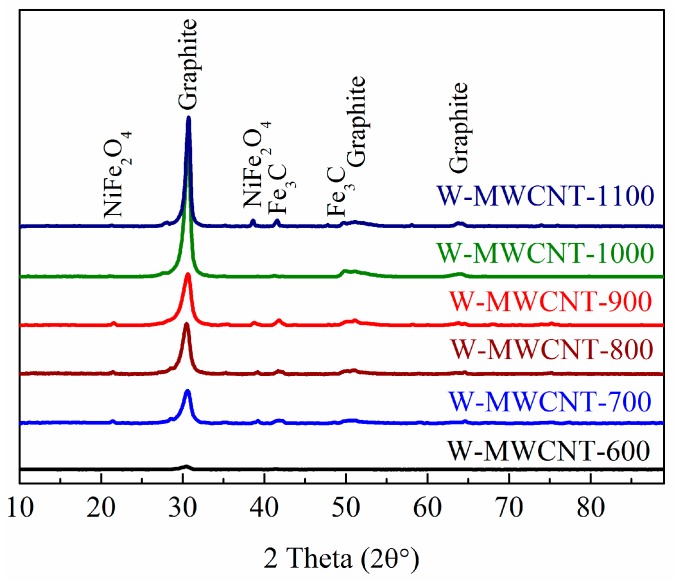
XRD patterns of the synthesized MWCNTs made from plastic centrifuge tubes made of polypropylene.

XRD analysis of the carbon products (Figure 7) revealed one dominant peak (30.8°), which can be assigned to the graphite (002) crystal plane with an internal plane spacing of 0.33 nm of MWCNTs. The products also showed three other peaks which are associated with graphite at 49.6, 52.1 and 64.2 degrees with unit lattice planes (100), (101) and (004), respectively (JCPDS: 00-012-0212) [47]. It is clearly observed that as the synthesis temperature increased, the crystallinity of the MWCNTs increased. At 1100 °C peaks were observed which can be assigned to iron carbide and nickel iron oxide (JCPDS: 00-003-0410) and JCPDS: 00-003-0875) [43,48].

### 2.3. Raman Spectra and TGA Analysis of the MWCNTs

**Figure 8:**
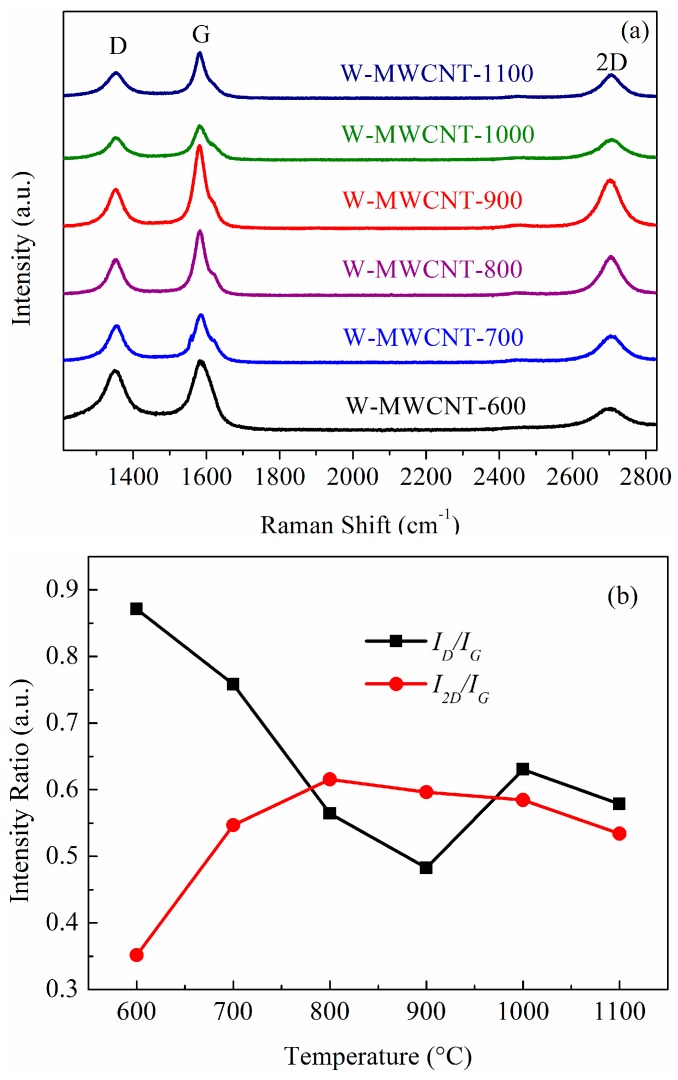
Raman spectral data for the W-MWCNTs (**a**) and as a function of growth temperature (**b**).

The crystallinity of the MWCNTs was studied using Raman spectroscopy (Figure 8a). There are three intense features in the spectra, which occur at ca. 1350 cm^−1^ (D Band), at ca. 1580 cm^−1^ (G Band) and at ca. 2700 cm^−1^ (2D Band) [49,50]. The D band arises from the A_1g_ in-plane breathing vibration and corresponds to a disordered sp^2^ hybridized carbon [51]. The G band is associated with the Raman-active E_2g_ in-plane vibration associated with the C–C tangential stretching mode and it is a characteristic of the graphitic phase, indicating the presence of crystalline graphitic MWCNTs [14,52]. The 2D band of the MWCNTs is associated with second-order zone-boundary phonons and provides information on defects in the MWCNTs [52]. As the synthesis temperature increased to 900 °C the G–band intensity increased, relative to that of the D–band intensity. It is known that the D– to G–band intensity ratio (*I*_D_/*I*_G_) is sensitive to structural defects in the MWCNTs; the lower the value, the more graphitic the carbon. It should be noted that the *I*_D_/*I*_G_ ratio consistently decreased until 900 °C and then increased at higher temperatures (Figure 8b). The lowest *I*_D_/*I*_G_ ratio (i.e., 0.48) was found for W-MWCNT-900. Similar reverse findings were noted for the *I*_2D_/*I*_G_ ratios as presented in Figure 8b [53]. We can conclude that the most graphitic carbons are grown at 900 °C. 

**Figure 9:**
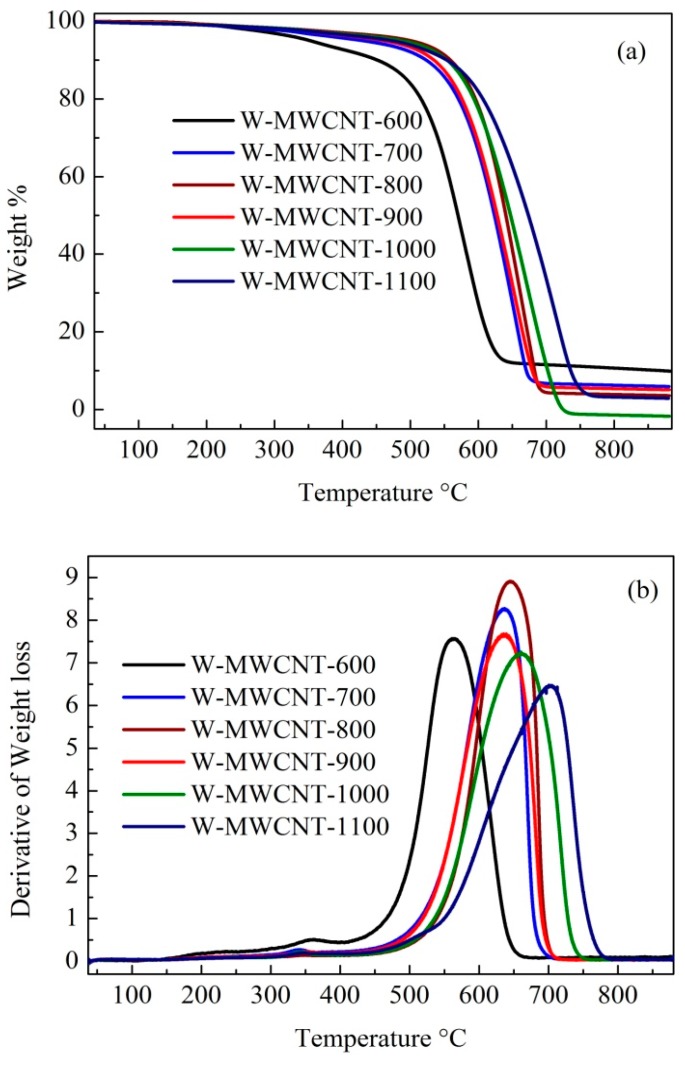
(**a**) Thermal profiles of the MWCNTs and (**b**) their derivate curves.

The oxidation of MWCNTs in air usually starts at T > 500 °C and prior to that any oxidation processes can be attributed to the presence of amorphous carbon or functional groups [54]. TGA data for the MWCNTs are presented in Figure 9. As can be seen in Figure 9a,b, W-MWCNT-600 showed two oxidation steps, firstly between 300 to 400 °C and then between 450 to 650 °C. It suggests that W-MWCNT-600 contains a minor amount of amorphous carbon, which decomposed first. The second oxidation step occurred at a temperature of close to 500 °C and suggests that the carbon is not highly graphitic or crystalline; consistent with the Raman and XRD data. W-MWCNT-700 synthesized at 700 °C also contained a small amount of amorphous carbon which was consistent with the Raman *I*_D_/*I*_G_ ratio. On the other hand, MWCNTs synthesized at higher temperatures (>800 °C) decomposed in air at T > 500 °C, suggesting a highly graphitic carbon. As the temperature of synthesis increased, the carbon decomposition temperature range shifted towards higher values. It can be noted from the decomposition profiles in Figure 9b that MWCNTs synthesized at a high temperature have a higher decomposition temperature range than those prepared at a lower temperature [55,56].

### 2.4. Mechanism of MWCNT Synthesis from Plastic Waste

**Figure 10:**
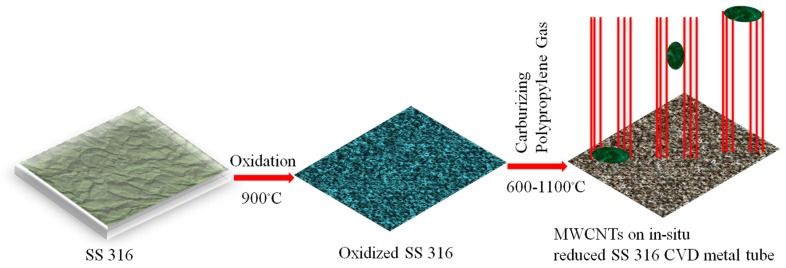
Mechanism of MWCNT growth using plastic waste and a SS 315 metal tube.

The carbon source used in the experiments was generated from plastic waste. The plastic waste readily decomposed cleanly into propylene in the reaction and this hydrocarbon was responsible for the MWCNT growth. This hydrocarbon is known to produce MWCNTs over metal catalysts [57]. The mechanism of MWCNT synthesis from hydrocarbons has been well documented and is known to occur either via a tip growth or a base-growth process involving a vapor-liquid-solid reaction [26,41]. There is still controversy as to aspects of the mechanism, for example, whether the carbon atoms generated from the carbon source dissolve in the bulk catalyst or react only in the top few carbon layers of a catalyst particle [58]. In the reaction reported in this study, the CVD reactor (SS 316 tube) acted as a catalyst for the MWCNT synthesis. Thus, the constituents of the reactor (wall) are responsible for the MWCNT growth. Similar CNT synthesis studies have been performed on SS 316 mesh and the results indicated that Fe (and Ni) were responsible for MWCNT formation [35]. Our results also indicated that the removal of the Cr covering of the steel is required for MWCNT growth, and our data are fully consistent with the studies by Levendis and co-workers [35]. TEM images indicated that metal catalysts were observed at the tip of the MWCNTs and inside the tubes (Figure 10) [57]. The data do not allow us to discriminate between a tip- and base-growth mechanism for CNT growth.

## 3. Materials and Methods

**Figure 11:**
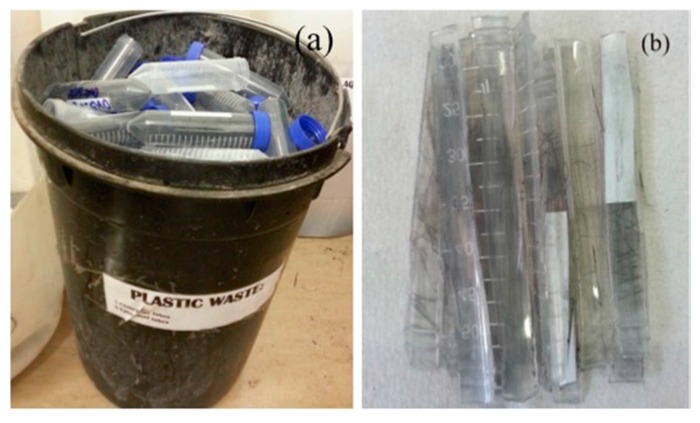
Optical images of (**a**) the laboratory-based used plastic centrifuge tubes and (**b**) the washed, dried and chopped used plastic centrifuge tubes.

**Figure 12:**
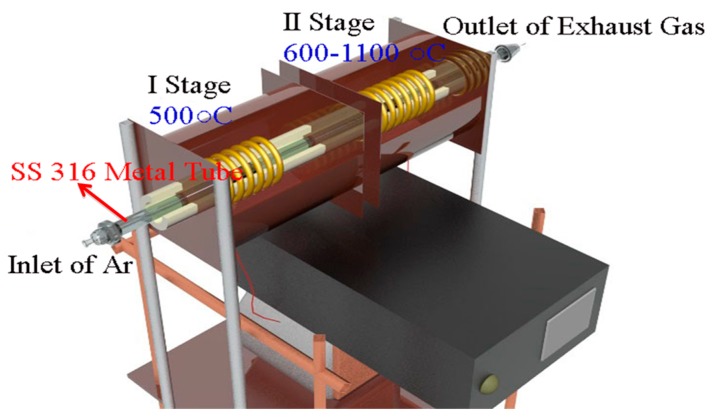
Schematic view of the two-stage SS 316 metal-tube-based CVD furnace used for the synthesis of W-MWCNTs from waste plastic centrifuge tubes made of polypropylene.

Waste centrifuge tubes were used as the hydrocarbon source. An optical photograph of the plastic waste is shown in Figure 11a. The plastic tubes were washed, dried and chopped into 0.5 cm × 8 cm pieces prior to use Figure 11b. Typically 1–2 g of plastic tube was used in every reaction. A reactor made of stainless steel (SS 316) was fabricated in the Physical Sciences Workshop, University of the Witwatersrand, Johannesburg, South Africa (Figure 12). The SS 316 metal tube was procured from Sandvik Materials Technology, Sweden, and was used as received. The SS 316 metal tube was placed in a two-stage CVD reactor i.e., a system containing two ovens that could be heated to two different temperatures. The plastic pieces were placed in the first stage of the reactor. Nitric acid (55%) was purchased from ACE, South Africa and used without any further purification or treatment.

### 3.1. Synthesis

**Figure 13:**
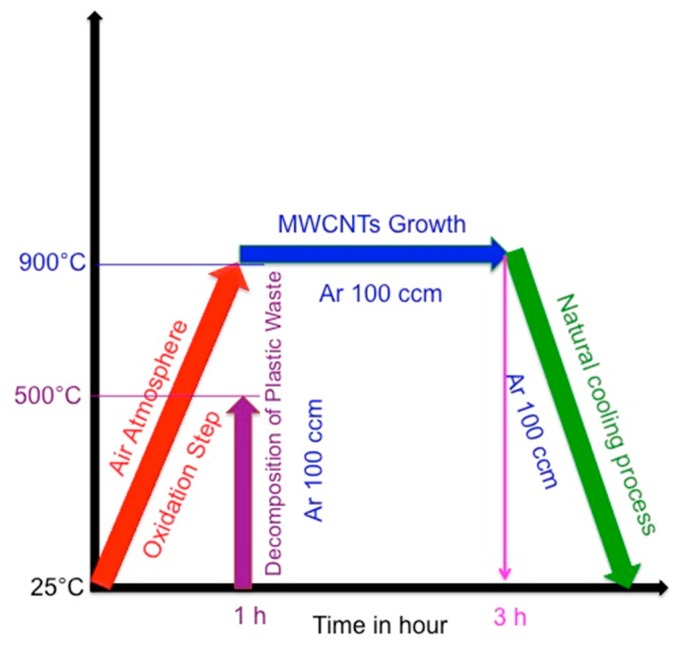
Pictorial representation of the W-MWCNT synthesis procedure using a steel reactor as catalyst and laboratory waste centrifuge tubes made of polypropylene as carbon source.

The plastic pieces were loaded into the first oven of the metal reactor at room temperature and the system was flushed with argon for 10 min. The second stage oven was then heated at 10 °C per minute under 100 sccm argon gas until the required reaction temperature had been reached (600, 700, 800, 900, 1000 and 1100 °C). Once this has been achieved, the first reactor stage was then heated at 10 °C per minute from room temperature to 500 °C. At this temperature, the plastic waste in the first oven was converted to propylene, which was used as the hydrocarbon source to make carbon materials by a catalytic decomposition process in the second stage of the reactor. The reaction was maintained at the required temperature for 2 h. After cooling of the CVD furnace, the black soot was recovered by scraping the black powder from the tube. The procedure used to make the carbon materials is summarized in Figure 13. The obtained materials were named according to the reaction temperature used viz. W-MWCNT-600, W-MWCNT-700, W-MWCNT-800, W-MWCNT-900, W-MWCNT-1000 and W-MWCNT-1100 for reactions performed at 600, 700, 800, 900, 1000 and 1100 °C, respectively, where the W stands for waste. Before a CVD run, the second stage of the metal tube SS 316 reactor was oxidized in static air at 900 °C for one hour at atmospheric pressure to remove any carbon residues and to oxidize the SS 316 reactor tube to enable it to act as a catalyst for further MWCNT synthesis.

### 3.2. Purification of W-MWCNTs

The purification and metal removal from the W-MWCNTs was done by acid treatment. Typically, 400 mg of W-MWCNTs was placed in a round bottom flask, 50 mL concentrated nitric acid was added and the suspension was refluxed for four hours at 120 °C. The mixture was filtered and washed copiously with distilled water until a near-neutral pH was reached (pH = 5–7). The yield of MWCNTs was determined by using Equation (1):

(1) $$\%\;\mathrm{Yield}\;\mathrm{of}\;W-\mathrm{MWCNTs}=\frac{\%\;\mathrm{Yield}\;\mathrm{from}\;\mathrm{TGA}\;\times \;\%\;\mathrm{Yield}\;\mathrm{with}\;\mathrm{metal}}{100}$$

where

(2) $$\%\;\mathrm{Yield}\;\mathrm{with}\;\mathrm{metal}=\frac{\mathrm{Obtained}\;\mathrm{carbon}\;\mathrm{after}\;\mathrm{CVD}}{\mathrm{Plastic}\;\mathrm{waste}\;\mathrm{used}\;\mathrm{for}\;\mathrm{CVD}}\;\times 100$$

### 3.3. Characterization

For the analysis of the bare and oxidized SS 316 tubes, a one cm^2^ section of tube was removed from the reactor using a pipe cutter and analyzed without any further polishing or treatment. A CAMECA SX5-FE EPMA (CAMECA, Gennevilliers CEDEX, France), equipped with five WDS (CAMECA, Gennevilliers CEDEX, France) detectors and twelve crystals to select X-ray wavelengths for specific elemental composition was used to map the distribution of iron (Fe-Kα), nickel (Ni-Kα), chromium (Cr-Kα) and oxygen (O-Kα) on the bare metal SS 316 tube and on the oxidized SS 316 tube. Elemental mapping was executed using a field emission guns-scanning electron microscopy (FEG-SEM) (CAMECA, Gennevilliers Cedex, France) linked to the EPMA-WDS. The FEG-SEM was operated at 15 kV. No sample preparation was required for elemental mapping using EPMA-WDS. The structural morphologies of the MWCNTs were observed by using TEM (Tecnai T12, FEI Company^TM^, Hillsboro, OR, USA) accelerated at a voltage of 120 kV and SEM (Nova Nanolab 600, FEI Company^TM^, Hillsboro, OR, USA) accelerated at a voltage of 30 kV. Samples for TEM measurements were prepared by dropping a methanolic suspension of MWCNTs onto a carbon film supported on a copper grid. Samples for SEM measurements were prepared by placing a carbon tape on top of a specimen holder and a small representative amount of fine powder of MWCNTs was dusted on the tape. Raman spectral measurements of W-MWCNTs were made with the 514.5 nm line of a Lexel Model 95 Second Harmonic Generation (SHG) argon ion laser and a LabRAM HR Raman spectrometer (Horiba, Kyoto, Japan) with an Olympus BX41 microscope attachment. The laser was directed onto the sample with a 100× objective and laser power at the sample was 0.4 mW. The beam spot size was a square of 10 micron × 10 micron, achieved by rastering the laser beam over a square with a DuoScan attachment. The backscattered light was dispersed via 600 lines/mm grating onto a liquid nitrogen cooled charge-coupled device (CCD) detector. The data acquisition software was LabSpec v5. The phase composition of the W-MWCNTs was determined by powder XRD analysis using a D2 phaser Bruker (Billerica, MA, USA) in Bragg–Brentano geometry with a Lynxeye detector using Co-Kα radiation at 30 kV and 10 mA. The scan range was 10° < 2*θ* < 90° in 0.040 steps, using a standard speed with an equivalent counting time of 1 s per step. The diffraction peaks were then compared with those of standard compounds reported in the Diffracto pulse evaluation package using the DIFFRAC.EVA V2-2014 (manufactured by Bruker AXS GmbH, Östliche Rheinbrückenstrasse, Karlsruhe, Germany) software package. TGA of W-MWCNTs were done using a Perkin Elmer simultaneous thermal analyzer (STA) 6000 (Waltham, MA, USA) under an air flow of 20 mL min^−1^ with a ramping temperature 10 °C min^−1^ in the temperature range from 35 to 900 °C. The sample weight of W-MWCNTs used for the TGA analysis was in the range 7.0 to 9.0 mg. The hyphenated technique of TGA with mass spectrometry (TGA-MS) was used (PFEIFFER Omnistar GSD 301 O (*m*/*z* < 200 a.m.u.) Asslar, Germany) to study the gases produced from the PP. The temperature ramping was done under an inert atmosphere (nitrogen). The ionization of the analyzed gas was performed using an axial beam ion source (100 eV). The ions, separated according to their mass-to-charge ratio, were detected by a Faraday collector. Evolved gases from the TGA chamber passed through a heated (160 °C) stainless steel capillary with an internal diameter of 0.15 mm. The coupling interface between the thermos-analyzer and the mass spectrometer operated simultaneously as a gas-input system and as a pressure reduction system. In order to eliminate cold points in the connecting line, the bottom of the thermos-analyzer was heated to approximately 160 °C.

## 4. Conclusions

Herein we report on the use of a plastic waste material (centrifuge tubes) to produce a value-added material, MWCNTs. The MWCNTs were made without the use of an external catalyst source by using a stainless-steel CVD reactor as the catalyst. Oxidation of the surface elements (in particular Cr) from the steel tube allowed for the other steel components (Fe, Ni) to act as catalysts to grow MWCNTs; this method thus takes advantage of the dusting process to produce a valuable carbon product. In another experiment, a non-oxidized SS 316 CVD reactor produced carbon nanofibers of micron thick diameter. In the thermal CVD synthesis studies at 600, 700, 800, 900, 1000 and 1100 °C the optimum synthesis temperature was 900 °C; at the optimum temperature of 900 °C the highest yield of MWCNTs was obtained and the best product crystallinity was produced. 

## Figures and Tables

**Table 1:** Content of SS 316 metal tube, which was used to make a two stage CVD furnace for the synthesis of MWCNTs using plastic waste as a carbon feed.

Elements	wt %
Carbon	0.08
Manganese	2.00
Phosphorus	0.045
Sulfur	0.030
Silicon	0.75
Chromium	16.00–18.00
Nickel	10.00–14.00
Molybdenum	2.00–3.00
Nitrogen	0.10
Iron	Balance

**Table 2:** Diameter and length of MWCNTs synthesized from PP.

Sample Name	Inner Diameter (nm)	Outer Diameter (nm)
W-MWCNT-600	8	28
W-MWCNT-700	9	30
W-MWCNT-800	9	33
W-MWCNT-900	10	35
W-MWCNT-1000	9	28
W-MWCNT-1100	8	32
